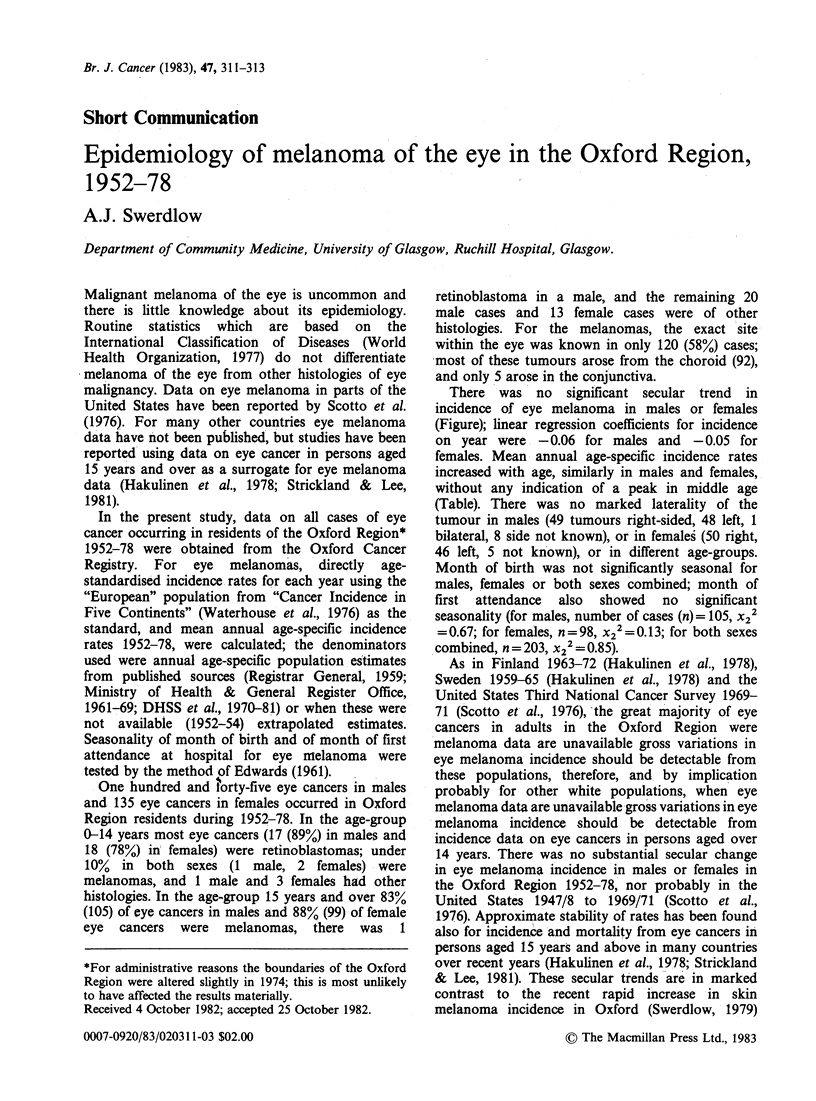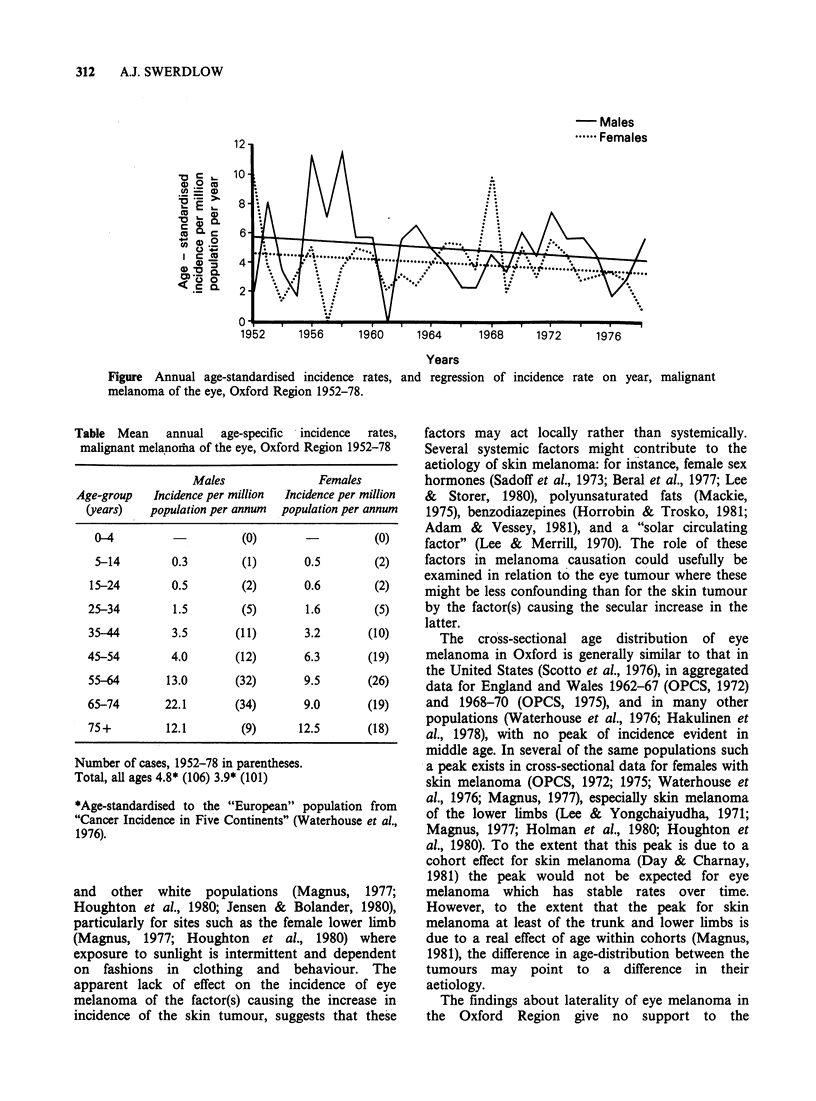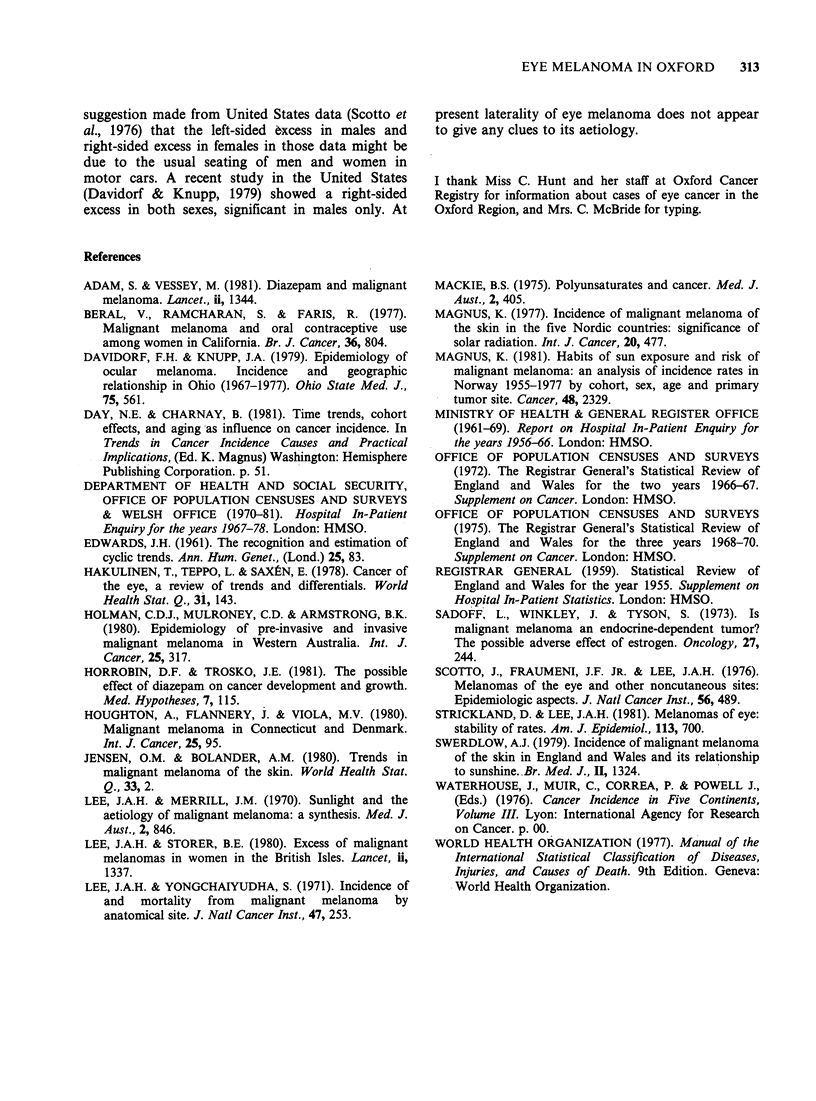# Epidemiology of melanoma of the eye in the Oxford Region, 1952-78.

**DOI:** 10.1038/bjc.1983.42

**Published:** 1983-02

**Authors:** A. J. Swerdlow


*Br. J. Cancer* (1983), **47**, 311–313

### Short Communication

### Epidemiology of melanoma of the eye in the Oxford Region, 1952–78

A.J. Swerdlow

*Department of Community Medicine, University of Glasgow, Ruchill Hospital, Glasgow.*

Malignant melanoma of the eye is uncommon and there is little knowledge about its epidemiology. Routine statistics which are based on the International Classification of Diseases (World Health Organization, 1977) do not differentiate melanoma of the eye from other histologies of eye malignancy. Data on eye melanoma in parts of the United States have been reported by Scotto *et al.* (1976). For many other countries eye melanoma data have not been published, but studies have been reported using data on eye cancer in persons aged 15 years and over as a surrogate for eye melanoma data (Hakulinen *et al.*, 1978; Strickland & Lee, 1981).

In the present study, data on all cases of eye cancer occurring in residents of the Oxford Region* 1952–78 were obtained from the Oxford Cancer Registry. For eye melanomas, directly age-standardised incidence rates for each year using the "European" population from "Cancer Incidence in Five Continents" (Waterhouse *et al.*, 1976) as the standard, and mean annual age-specific incidence rates 1952–78, were calculated; the denominators used were annual age-specific population estimates from published sources (Registrar General, 1959; Ministry of Health & General Register Office, 1961–69; DHSS *et al.*, 1970–81) or when these were not available (1952–54) extrapolated estimates. Seasonality of month of birth and of month of first attendance at hospital for eye melanoma were tested by the method of Edwards (1961).

One hundred and forty-five eye cancers in males and 135 eye cancers in females occurred in Oxford Region residents during 1952–78. In the age-group 0–14 years most eye cancers (17 (89%) in males and 18 (78%) in females) were retinoblastomas; under 10% in both sexes (1 male, 2 females) were melanomas, and 1 male and 3 females had other histologies. In the age-group 15 years and over 83% (105) of eye cancers in males and 88% (99) of female eye cancers were melanomas, there was 1 retinoblastoma in a male, and the remaining 20 male cases and 13 female cases were of other histologies. For the melanomas, the exact site within the eye was known in only 120 (58%) cases; most of these tumours arose from the choroid (92), and only 5 arose in the conjunctiva.

There was no significant secular trend in incidence of eye melanoma in males or females (Figure); linear regression coefficients for incidence on year were −0.06 for males and −0.05 for females. Mean annual age-specific incidence rates increased with age, similarly in males and females, without any indication of a peak in middle age (Table). There was no marked laterality of the tumour in males (49 tumours right-sided, 48 left, 1 bilateral, 8 side not known), or in females (50 right, 46 left, 5 not known), or in different age-groups. Month of birth was not significantly seasonal for males, females or both sexes combined; month of first attendance also showed no significant seasonality (for males, number of cases $(n)=105$, $x_2{}^2=0.67$; for females, $n=98$, $x_2{}^2=0.13$; for both sexes combined, $n=203$, $x_2{}^2=0.85$).

As in Finland 1963–72 (Hakulinen *et al.*, 1978), Sweden 1959–65 (Hakulinen *et al.*, 1978) and the United States Third National Cancer Survey 1969–71 (Scotto *et al.*, 1976), the great majority of eye cancers in adults in the Oxford Region were melanoma data are unavailable gross variations in eye melanoma incidence should be detectable from these populations, therefore, and by implication probably for other white populations, when eye melanoma data are unavailable gross variations in eye melanoma incidence should be detectable from incidence data on eye cancers in persons aged over 14 years. There was no substantial secular change in eye melanoma incidence in males or females in the Oxford Region 1952–78, nor probably in the United States 1947/8 to 1969/71 (Scotto *et al.*, 1976). Approximate stability of rates has been found also for incidence and mortality from eye cancers in persons aged 15 years and above in many countries over recent years (Hakulinen *et al.*, 1978; Strickland & Lee, 1981). These secular trends are in marked contrast to the recent rapid increase in skin melanoma incidence in Oxford (Swerdlow, 1979)

*For administrative reasons the boundaries of the Oxford Region were altered slightly in 1974; this is most unlikely to have affected the results materially.

Received 4 October 1982; accepted 25 October 1982.

0007-0920/83/020311-03 $02.00
© The Macmillan Press Ltd., 1983

**Figure** Annual age-standardised incidence rates, and regression of incidence rate on year, malignant melanoma of the eye, Oxford Region 1952–78.

**Table** Mean annual age-specific incidence rates, malignant melanoma of the eye, Oxford Region 1952–78

| *Age-group (years)* | *Males Incidence per million population per annum* | | *Females Incidence per million population per annum* | |
|---|---|---|---|---|
| 0–4 | — | (0) | — | (0) |
| 5–14 | 0.3 | (1) | 0.5 | (2) |
| 15–24 | 0.5 | (2) | 0.6 | (2) |
| 25–34 | 1.5 | (5) | 1.6 | (5) |
| 35–44 | 3.5 | (11) | 3.2 | (10) |
| 45–54 | 4.0 | (12) | 6.3 | (19) |
| 55–64 | 13.0 | (32) | 9.5 | (26) |
| 65–74 | 22.1 | (34) | 9.0 | (19) |
| 75+ | 12.1 | (9) | 12.5 | (18) |

Number of cases, 1952–78 in parentheses.
Total, all ages 4.8* (106) 3.9* (101)

*Age-standardised to the "European" population from "Cancer Incidence in Five Continents" (Waterhouse *et al.*, 1976).

and other white populations (Magnus, 1977; Houghton *et al.*, 1980; Jensen & Bolander, 1980), particularly for sites such as the female lower limb (Magnus, 1977; Houghton *et al.*, 1980) where exposure to sunlight is intermittent and dependent on fashions in clothing and behaviour. The apparent lack of effect on the incidence of eye melanoma of the factor(s) causing the increase in incidence of the skin tumour, suggests that these factors may act locally rather than systemically. Several systemic factors might contribute to the aetiology of skin melanoma: for instance, female sex hormones (Sadoff *et al.*, 1973; Beral *et al.*, 1977; Lee & Storer, 1980), polyunsaturated fats (Mackie, 1975), benzodiazepines (Horrobin & Trosko, 1981; Adam & Vessey, 1981), and a "solar circulating factor" (Lee & Merrill, 1970). The role of these factors in melanoma causation could usefully be examined in relation to the eye tumour where these might be less confounding than for the skin tumour by the factor(s) causing the secular increase in the latter.

The cross-sectional age distribution of eye melanoma in Oxford is generally similar to that in the United States (Scotto *et al.*, 1976), in aggregated data for England and Wales 1962–67 (OPCS, 1972) and 1968–70 (OPCS, 1975), and in many other populations (Waterhouse *et al.*, 1976; Hakulinen *et al.*, 1978), with no peak of incidence evident in middle age. In several of the same populations such a peak exists in cross-sectional data for females with skin melanoma (OPCS, 1972; 1975; Waterhouse *et al.*, 1976; Magnus, 1977), especially skin melanoma of the lower limbs (Lee & Yongchaiyudha, 1971; Magnus, 1977; Holman *et al.*, 1980; Houghton *et al.*, 1980). To the extent that this peak is due to a cohort effect for skin melanoma (Day & Charnay, 1981) the peak would not be expected for eye melanoma which has stable rates over time. However, to the extent that the peak for skin melanoma at least of the trunk and lower limbs is due to a real effect of age within cohorts (Magnus, 1981), the difference in age-distribution between the tumours may point to a difference in their aetiology.

The findings about laterality of eye melanoma in the Oxford Region give no support to the suggestion made from United States data (Scotto *et al.*, 1976) that the left-sided excess in males and right-sided excess in females in those data might be due to the usual seating of men and women in motor cars. A recent study in the United States (Davidorf & Knupp, 1979) showed a right-sided excess in both sexes, significant in males only. At present laterality of eye melanoma does not appear to give any clues to its aetiology.

I thank Miss C. Hunt and her staff at Oxford Cancer Registry for information about cases of eye cancer in the Oxford Region, and Mrs. C. McBride for typing.

**References**

ADAM, S. & VESSEY, M. (1981). Diazepam and malignant melanoma. *Lancet.*, **ii**, 1344.

BERAL, V., RAMCHARAN, S. & FARIS, R. (1977). Malignant melanoma and oral contraceptive use among women in California. *Br. J. Cancer*, **36**, 804.

DAVIDORF, F.H. & KNUPP, J.A. (1979). Epidemiology of ocular melanoma. Incidence and geographic relationship in Ohio (1967–1977). *Ohio State Med. J.*, **75**, 561.

DAY, N.E. & CHARNAY, B. (1981). Time trends, cohort effects, and aging as influence on cancer incidence. In *Trends in Cancer Incidence Causes and Practical Implications*, (Ed. K. Magnus) Washington: Hemisphere Publishing Corporation. p. 51.

DEPARTMENT OF HEALTH AND SOCIAL SECURITY, OFFICE OF POPULATION CENSUSES AND SURVEYS & WELSH OFFICE (1970–81). *Hospital In-Patient Enquiry for the years 1967–78*. London: HMSO.

EDWARDS, J.H. (1961). The recognition and estimation of cyclic trends. *Ann. Hum. Genet.*, (Lond.) **25**, 83.

HAKULINEN, T., TEPPO, L. & SAXÉN, E. (1978). Cancer of the eye, a review of trends and differentials. *World Health Stat. Q.*, **31**, 143.

HOLMAN, C.D.J., MULRONEY, C.D. & ARMSTRONG, B.K. (1980). Epidemiology of pre-invasive and invasive malignant melanoma in Western Australia. *Int. J. Cancer*, **25**, 317.

HORROBIN, D.F. & TROSKO, J.E. (1981). The possible effect of diazepam on cancer development and growth. *Med. Hypotheses*, **7**, 115.

HOUGHTON, A., FLANNERY, J. & VIOLA, M.V. (1980). Malignant melanoma in Connecticut and Denmark. *Int. J. Cancer*, **25**, 95.

JENSEN, O.M. & BOLANDER, A.M. (1980). Trends in malignant melanoma of the skin. *World Health Stat. Q.*, **33**, 2.

LEE, J.A.H. & MERRILL, J.M. (1970). Sunlight and the aetiology of malignant melanoma: a synthesis. *Med. J. Aust.*, **2**, 846.

LEE, J.A.H. & STORER, B.E. (1980). Excess of malignant melanomas in women in the British Isles. *Lancet*, **ii**, 1337.

LEE, J.A.H. & YONGCHAIYUDHA, S. (1971). Incidence of and mortality from malignant melanoma by anatomical site. *J. Natl Cancer Inst.*, **47**, 253.

MACKIE, B.S. (1975). Polyunsaturates and cancer. *Med. J. Aust.*, **2**, 405.

MAGNUS, K. (1977). Incidence of malignant melanoma of the skin in the five Nordic countries: significance of solar radiation. *Int. J. Cancer*, **20**, 477.

MAGNUS, K. (1981). Habits of sun exposure and risk of malignant melanoma: an analysis of incidence rates in Norway 1955–1977 by cohort, sex, age and primary tumor site. *Cancer*, **48**, 2329.

MINISTRY OF HEALTH & GENERAL REGISTER OFFICE (1961–69). *Report on Hospital In-Patient Enquiry for the years 1956–66*. London: HMSO.

OFFICE OF POPULATION CENSUSES AND SURVEYS (1972). The Registrar General's Statistical Review of England and Wales for the two years 1966–67. *Supplement on Cancer*. London: HMSO.

OFFICE OF POPULATION CENSUSES AND SURVEYS (1975). The Registrar General's Statistical Review of England and Wales for the three years 1968–70. *Supplement on Cancer*. London: HMSO.

REGISTRAR GENERAL (1959). Statistical Review of England and Wales for the year 1955. *Supplement on Hospital In-Patient Statistics*. London: HMSO.

SADOFF, L., WINKLEY, J. & TYSON, S. (1973). Is malignant melanoma an endocrine-dependent tumor? The possible adverse effect of estrogen. *Oncology*, **27**, 244.

SCOTTO, J., FRAUMENI, J.F. JR. & LEE, J.A.H. (1976). Melanomas of the eye and other noncutaneous sites: Epidemiologic aspects. *J. Natl Cancer Inst.*, **56**, 489.

STRICKLAND, D. & LEE, J.A.H. (1981). Melanomas of eye: stability of rates. *Am. J. Epidemiol.*, **113**, 700.

SWERDLOW, A.J. (1979). Incidence of malignant melanoma of the skin in England and Wales and its relationship to sunshine. *Br. Med. J.*, **II**, 1324.

WATERHOUSE, J., MUIR, C., CORREA, P. & POWELL J., (Eds.) (1976). *Cancer Incidence in Five Continents, Volume III*. Lyon: International Agency for Research on Cancer. p. 00.

WORLD HEALTH ORGANIZATION (1977). *Manual of the International Statistical Classification of Diseases, Injuries, and Causes of Death*. 9th Edition. Geneva: World Health Organization.