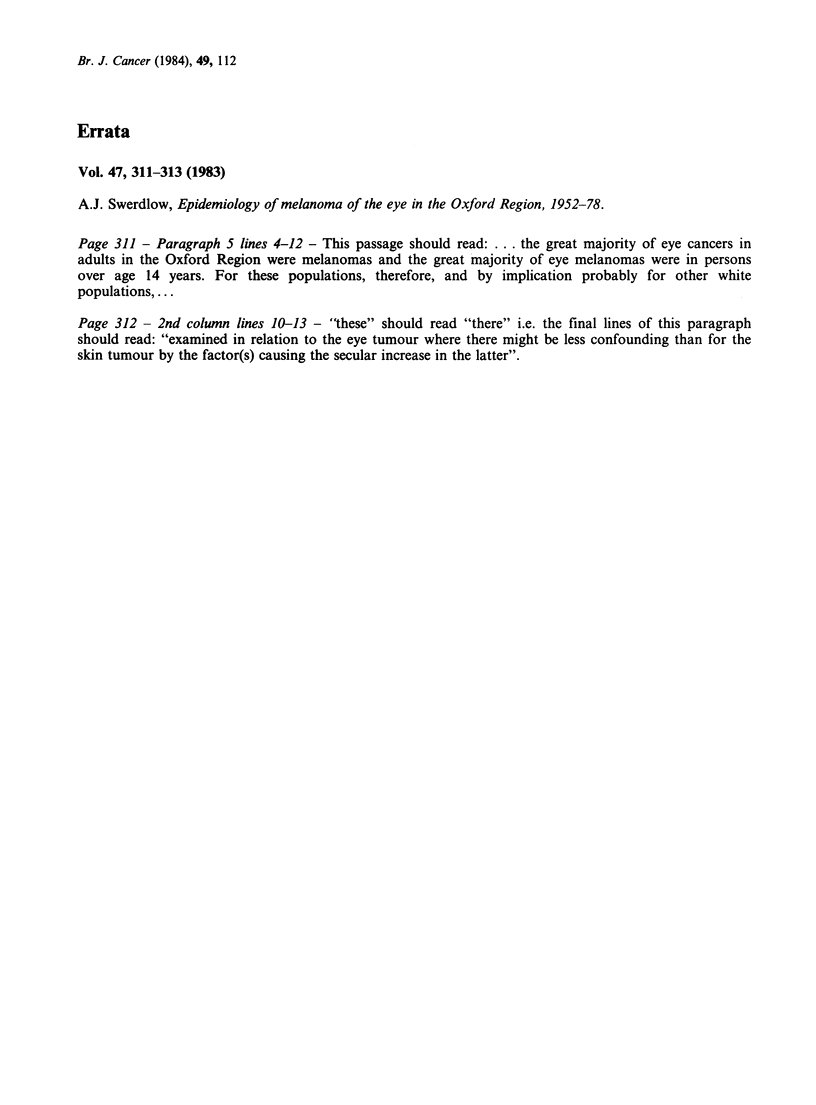# Errata

**Published:** 1984-01

**Authors:** 


*Br. J. Cancer* (1984), **49**, 112

### Errata

**Vol. 47, 311–313 (1983)**

A.J. Swerdlow, *Epidemiology of melanoma of the eye in the Oxford Region, 1952–78.*

*Page 311 – Paragraph 5 lines 4–12* – This passage should read: . . . the great majority of eye cancers in adults in the Oxford Region were melanomas and the great majority of eye melanomas were in persons over age 14 years. For these populations, therefore, and by implication probably for other white populations, . . .

*Page 312 – 2nd column lines 10–13* – "these" should read "there" i.e. the final lines of this paragraph should read: "examined in relation to the eye tumour where there might be less confounding than for the skin tumour by the factor(s) causing the secular increase in the latter".